# Qualitative correlation between postoperatively increased vertical dimension and mandibular position in skeletal class III using partial-least-square path modeling

**DOI:** 10.1186/s40902-017-0114-4

**Published:** 2017-06-05

**Authors:** Na-Ri Kim, Soo-Byung Park, Jihyun Lee, Youn-Kyung Choi, Sang Min Shin, Yong-Seok Choi, Yong-Il Kim

**Affiliations:** 1 Department of Orthodontics, Pusan National University Hospital, Busan, South Korea; 2 Department of Management Information Systems, College of Business, Dong-A University, Busan, South Korea; 3 Department of Statistics, College of Natural Science, Pusan National University, Busan, South Korea; 4 Institute of translational dental sciences, Pusan National University, Busan, South Korea; 5 Geumoro 20, Mulgeumeup, Yangsan 626-787 South Korea

**Keywords:** Surgery-first approach, Vertical dimension, Mandibular position, Pathway, Modeling

## Abstract

**Background:**

This study constructed a partial-least-square path-modeling (PLS-PM) model and found the pathway by which the postsurgical vertical dimension (VD) affects the extent of the final mandibular setback on the B point at the posttreatment stage for the skeletal class III surgery-first approach (SFA).

**Methods:**

This study re-analyzed the data from the retrospective study by Lee et al. on 40 patients with skeletal class III bimaxillary SFA. Variables were obtained from cone beam computed tomography (CBCT)-generated cephalograms. Authors investigated all variables at each time point to build a PLS-PM model to verify the effect of the VD on the final setback of the mandible.

**Results:**

From PLS-PM, an increase in VD_10_ was found to decrease the absolute value of the final setback amount of the mandible, which reflects the postsurgical physiological responses to both surgery and orthodontic treatment, which, in turn, can be interpreted as an increase in postoperative mandibular changes.

**Conclusions:**

To resolve the issue of collinear cephalometric data, the present study adopted PLS-PM to assess the orthodontic treatment. From PLS-PM, it was able to summarize the effect of increased postsurgery occlusal vertical dimension on the increased changeability of the B point position at the posttreatment stage.

## Background

Many clinical studies in dental research have used multiple regression analysis to find correlations between the predictor and the response variables. However, one possible problem with this approach is the collinearity between or among predictors. In statistics, when there are more than two predictors that are highly correlated, it is called collinearity [1, 2]. The interpretation of the statistical results from multiple regression models might be distorted by collinearity. The variables in research are mathematically coupled, so they are statistically collinear [2, 3]. In the fields of orthodontic and orthognathic surgery, several anatomic landmarks’ variables, obtained from each part of the craniofacial structure, might be mutually related, because each segment moves simultaneously with those landmarks during orthodontic or orthognathic procedures. Therefore, some variables from orthodontic research should be considered to detect collinearity.

Partial-least-square (PLS) modeling has been used for success factor studies in marketing and for estimating several satisfaction index models [4, 5]. PLS modeling is also often used as an alternative to conventional statistical modeling methods. Unlike conventional modeling methods, which rely on covariance decomposition, PLS is a variance-based method that does not carry the covariance assumptions. Therefore, PLS is less sensitive to the problems arising from collinearity [6, 7].

Tu and co-workers attempted to resolve the collinearity problems in periodontal research by using PLS regression and applied PLS path modeling to describe the relationship between baseline characteristics of infrabony lesions and the treatment outcomes [3]. In the fields of orthodontics and orthognathic, a few studies have considered the problems induced from the assessment of treatment outcomes by PLS path modeling (PLS-PM) [8].

**Fig. 1:**
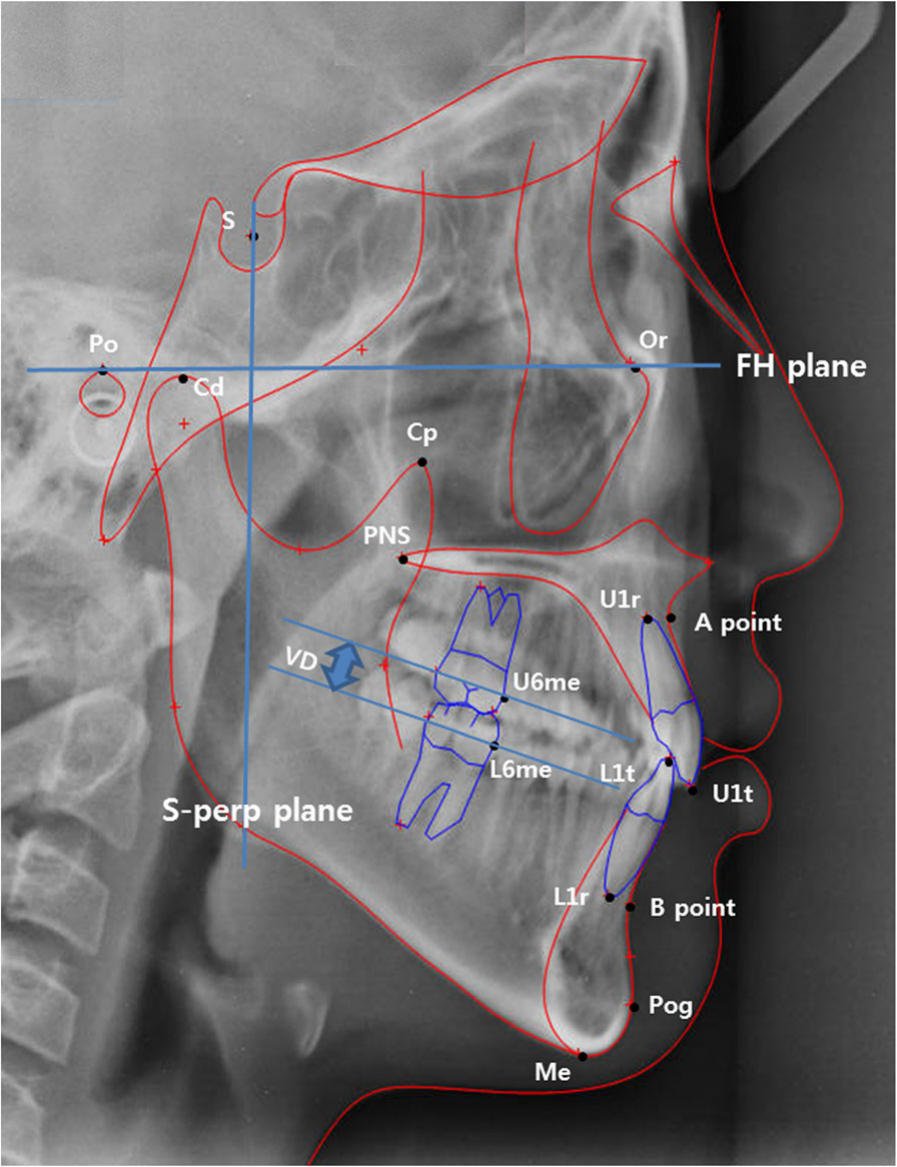
Landmarks and linear measurements. The distances were measured from the Frankfort-horizontal (FH) plane (*vertical*) and the Sella-perpendicular (S-perp) plane (*horizontal*) to each landmark. Condylion (*Cd*): most superior point of condyle head. Coronoid process (*Cp*): tip of coronoid process. *U1t* and *L1t*: upper and lower 1^st^ incisor tip. *U1r* and *L1r*: upper and lower 1^st^ incisor root apex. *U6mc* and *L6mc*: upper and lower 1^st^ molar mesial contact. Occlusal vertical dimension (*VD*): distance between mesial contact points of upper-right 1^st^ molar and lower-right 1^st^ molar as projected onto the S-perp plane

Our study was inspired by a clinical observation that a longer postsurgical (*T*_1_) occlusal vertical dimension (VD) was related to a more severe relapse for the class III surgery-first approach (SFA) cases. The following clinical questions were raised: What variables (cone beam computed tomography (CBCT)-generated cephalometric measurements) account for the final mandibular setback movement? How does postsurgical VD affect it? To answer these questions, we calculated and re-analyzed the variables for occlusal VD, represented as the distance between the mesial contact points of the upper-right 1^st^ molar and the lower-right 1^st^ molar, as projected onto the S-perpendicular plane (Fig. 1). However, if upper and lower segments move after orthognathic surgery, variables of each segment might influence the VD variable. Therefore, each variable was coupled and correlated. These collinearity problems should be resolved for investigation of the relationships between and among the measurement variables. In this study, therefore, PLS-PM was applied.

The aim of this study was to re-analyze collinear data from the cephalometric study by Lee et al. [9] using PLS-PM, to construct a PLS-PM model, and finally to find the pathway whereby the postsurgical vertical dimension (VD at *T*_1_) affects the extent of the final mandibular setback on the B point at the posttreatment stage (*T*_2_) for the skeletal class III surgery-first approach (SFA).

## Methods

We re-analyzed the data from the retrospective study by Lee and co-workers [9] on 40 patients with skeletal class III SFA (16 males, 24 females; mean age, 22.6 ± 4.0 years) in the Department of Oral and Maxillofacial Surgery and the Department of Orthodontics, at Pusan National University Hospital. In accordance with SFA procedures, all patients received LeFort I osteotomy and mandibular setback sagittal split ramus osteotomy (SSRO). This study was reviewed and approved by the Institutional Review Board of Pusan National University Hospital (E-2014021).

Variables were obtained from the 2-dimensional coordinates of each skeletal landmark, which were extracted by the superimposition method from CBCT-generated cephalograms (DCT pro; Vatech Co., Seoul, Korea) [10] (Fig. 1, Table 1). The imaging software (Ondemand3D; Cybermed Co., Seoul, Korea) created CBCT-generated half-cephalograms, superimposed on the anterior cranial base, and digitized by software (V-ceph6.0; Osstem Co., Seoul, Korea).

**Table 1:** Model-relevant landmarks, planes, distances, and indicators

Terms	Definitions
Landmarks	
B point (B)	Innermost curvature from chin to alveolar bone junction
Menton (Me)	Lowest point on symphysis of mandible
Pogonion (Pog)	Most anterior point on contour of chin
A point (A)	Innermost curvature from maxillary anterior nasal spine to crest of maxillary alveolar process
Coronoid process (Cp)	Tip of coronoid process
Planes	
Frankfurt-horizontal (FH) plane	Plane formed by right Po and both sides of Or
Midsagittal reference (MSR) plane	Perpendicular to FH plane and passing through Na and Ba
S-perpendicular plane (S-perp)	Perpendicular to FH and MSR planes and passing through Sella
Distances	
B point to S-perp (B^s^)	Distance from B point to S-perpendicular plane
Menton to S-perp (Me^s^)	Distance from menton to S-perpendicular plane
Pogonion to FH plane (Pog^f^)	Distance from pogonion to FH plane
A point to S-perp (A^*s*^)	Distance from A point to S-perpendicular plane
Coronoid process to FH plane (Cp^f^)	Distance from tip of coronoid process to FH plane
Occlusal vertical distance (VD)	Distance from mesial contact of upper 1st molar to that of lower 1st molar
Indicators	
Final setback of B point $\left({\mathrm{B}}_{20}^{\mathrm{s}}\right)$	B^s^ changes from *T*_0_ to *T*_2_, combined effects of surgery and orthodontic treatment over presurgical (*T*_0_) to posttreatment period (*T*_2_)
Surgical setback of B point $\left({\mathrm{B}}_{10}^{\mathrm{s}}\right)$	B^s^ changes from *T*_0_ to *T*_1_, surgical effects measured over presurgical (*T*_0_) to postsurgical period (*T*_1_)
Surgical setback of menton $\left({\mathrm{Me}}_{10}^{\mathrm{s}}\right)$	Me^s^ changes from *T*_0_ to *T*_1_, surgical effects measured over presurgical (*T*_0_) to postsurgical period (*T*_1_)
Surgical upward movement of pogonion $\left({\mathrm{Pog}}_{10}^{\mathrm{f}}\right)$	Pog^f^ changes from *T*_0_ to *T*_1_, surgical effects measured over presurgical (*T*_0_) to postsurgical period (*T*_1_)
Postsurgical horizontal position of A point $\left({\mathrm{A}}_1^{\mathrm{s}}\right)$	Distance from A point to S-perpendicular plane measured at *T*_1_
Postsurgical vertical position of coronoid process $\left({\mathrm{Cp}}_1^{\mathrm{f}}\right)$	Distance from tip of coronoid process to FH plane measured at *T*_1_

From the horizontal (FH plane) and vertical reference planes (S-perpendicular plane), variables were obtained at preoperative, postoperative, and posttreatment (*T*_0_, *T*_1_, and *T*_2_) time points and calculated along with the corresponding increment or decrement in such distances over the periods of *T*_0_ to *T*_1_ (∆*T*_1_ − *T*_0_) and *T*_1_ to *T*_2_ (∆*T*_2_ − *T*_1_), respectively. For the horizontal position of the B point, the change in its distance from the S-perpendicular plane over the period *T*_0_ to *T*_2_ (∆*T*_2_ − *T*_0_) was calculated (Table 2).

**Table 2:** Variables tested for multivariate regression model

		*T*_0_	*T*_1_	*T*_2_
Horizontal parameters				
Maxilla	A^s^	62.5 (4.4)	63.0 (4.5)	62.6 (4.8)
	PNS^s^	20.0 (3.1)	20.3 (3.1)	20.8 (3.1)
Distal segment of mandible	B^s^	66.6 (7.0)	58.2 (6.7)	59.0 (7.3)
	Pog^s^	68.3 (7.5)	61.1 (7.4)	62.0 (7.9)
	Me^s^	62.1 (7.4)	54.4 (7.0)	55.2 (8.1)
Proximal segment of mandible	Cd^s^	−12.0 (3.3)	−11.2 (3.4)	−12.1 (3.3)
	Cp^s^	25.1 (3.7)	25.7 (3.8)	25.4 (3.7)
Dental parameters	U1^s^	69.3 (5.0)	69.1 (5.2)	68.8 (5.6)
	U1r^s^	58.3 (4.5)	58.9 (4.8)	58.8 (5.1)
	L1^s^	70.7 (6.6)	62.1 (5.6)	64.5 (5.8)
	L1r^s^	63.7 (7.2)	55.4 (6.8)	55.4 (7.3)
	U6^s^	43.3 (4.2)	43.5 (4.7)	42.9 (4.9)
	L6^s^	47.1 (17.6)	38.8 (14.4)	40.2 (15.4)
Vertical parameters				
Maxilla	A^f^	31.2 (3.3)	28.2 (3.5)	27.8 (3.4)
	PNS^f^	24.9 (2.8)	20.2 (3.9)	20.0 (3.8)
Distal segment of mandible	B^f^	77.9 (6.5)	75.0 (5.4)	75.2 (7.5)
	Pog^f^	91.8 (8.0)	88.1 (6.6)	86.0 (7.1)
	Me^f^	98.3 (8.1)	94.3 (6.6)	92.6 (7.3)
Proximal segment of mandible	Cd^f^	2.6 (2.0)	3.4 (2.4)	2.5 (2.5)
	Cp^f^	9.8 (4.8)	11.3 (4.6)	8.6 (4.7)
Dental parameters	U1^f^	55.2 (5.1)	53.4 (4.7)	53.5 (4.7)
	U1r^f^	34.3 (4.2)	31.3 (3.8)	31.3 (3.8)
	L1^f^	55.6 (5.2)	52.4 (4.3)	51.1 (4.7)
	L1r^f^	74.8 (6.1)	71.5 (5.1)	70.3 (5.5)
	U6^f^	47.6 (4.4)	44.1 (4.3)	43.5 (4.5)
	L6^f^	54.0 (18.6)	50.7 (17.3)	48.8 (16.6)
Occlusal vertical dimension	VD	11.8 (3.3)	9.9 (1.9)	8.2 (1.3)

All variables at each time point were investigated for construction of the PLS-PM for verification of the effect of the VD on the final setback of the mandible. Using PLS-PM, we tested various possible pathways by which the VD may affect the final mandibular setback extent. In the PLS-PM framework [11], the path modeling is composed of the measurement model (the outer model) and the structural model (the inner model) [12], wherein the predictor variables (${\mathrm{B}}_{10}^{\mathrm{s}},\kern0.5em {\mathrm{Me}}_{10}^{\mathrm{s}},\kern0.5em {\mathrm{A}}_1^{\mathrm{s}},\kern0.5em {\mathrm{Cp}}_1^{\mathrm{f}},\kern0.5em \mathrm{V}{\mathrm{D}}_{10}$ from the Lee study [9]) consisted of the measurement components or, equivalently, the manifest variables (MVs). Meanwhile, the latent variables (LVs) LV_1_, LV_10_, LV_overall_, and LV_setback,_ which conceptualize the various postsurgical physiologic responses to surgery and orthodontic treatment that ultimately influence the final setback amount of the mandible, were considered in following the protocol of path modeling [10]. As for the outer model, wherein the causal relationships between a latent variable and manifest variables as their observed indicators are specified [13] as a formative outer model [12], a latent variable is defined as a linear combination of its corresponding manifest variables [11, 12]. The inner model is composed of LV_1_, LV_10_, LV_overall_, and LV_setback_, wherein the pattern of the relationship of an LV to other LVs can be expressed as a linear combination of those LVs that are relevant to it. In particular, LV_setback_, which is equivalent to the absolute value of the final mandibular setback extent, was expressed as a linear combination of all other LVs. Since there is as yet no generally accepted model-fit index in the PLS-PM, a global criterion for goodness-of-fit (GoF) suggested by Tenenhaus et al. [6] was used to evaluate the prediction performance of the model, along with the *R*-square values observed within the framework of the inner model. For statistical analysis of the multivariate regression and the PLS-PM, the language R (Vienna, Austria), an open-source software program for statistical computation, was used.

## Results

**Fig. 2:**
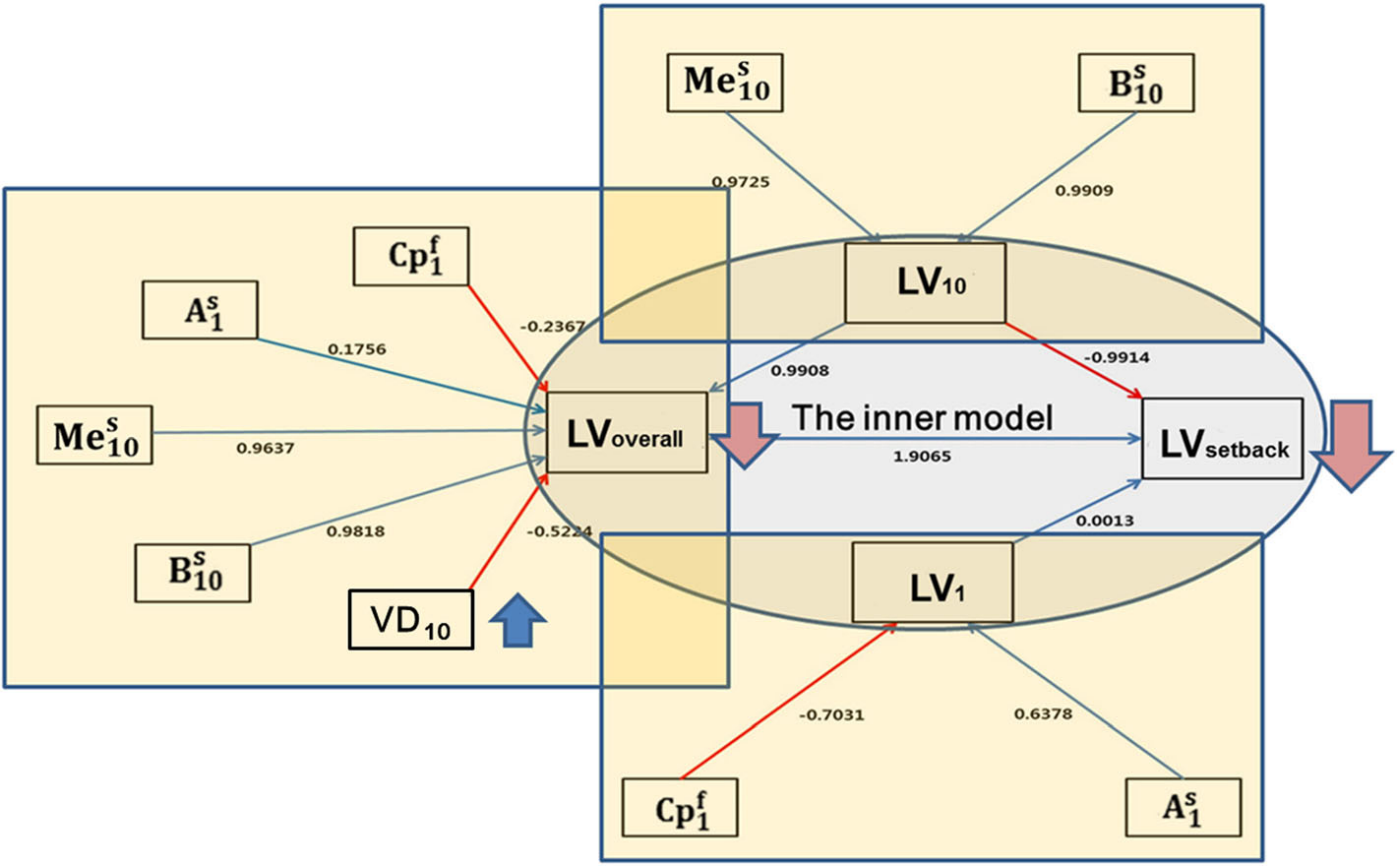
Outer and inner models derived via partial-least-square path modeling (PLS-PM). The three larger rectangles represent the outer models, wherein the latent variables (LVs) LV_overall_, LV_10_, and LV_1_ are formed by their corresponding manifest variables (MVs), represented by the *smaller rectangles*. The *ovoid* in the middle represents the quantitative interrelationship between LVs representing the inner model. The *vertical arrows* indicate the effect of VD on the LV_setback_ or on the amount of mandibular setback (i.e., the absolute value of the final setback of B point), indicating that an increase in VD_10_ (*blue arrow*) results in a decrease in the final mandibular setback amount measured at *T*_2_ (*red arrow*), observed as a more severe relapse

In the present study, a quantitatively analytic method of PLS-PM was adopted to verify the effect of VD on the extent of final mandibular setback. In the PLS-PM, ${\mathrm{B}}_{10}^{\mathrm{s}},\kern0.5em {\mathrm{Me}}_{10}^{\mathrm{s}},\kern0.5em {\mathrm{A}}_1^{\mathrm{s}}$ and ${\mathrm{Cp}}_1^{\mathrm{f}}$, along with VD_10_, consisted of MVs, which are observable values by definition in the PLS-PM framework (Table 3, Fig. 2), as well as the LVs LV_1_, LV_10_, LV_overall,_ and LV_setback_ and their presumed interrelationships (Tables 3, 4 and Fig. 2). As for the outer model, the MVs of ${\mathrm{B}}_{10}^{\mathrm{s}}$ and ${\mathrm{Me}}_{10}^{\mathrm{s}}$ were presumed to cause LV_10_, while those of ${\mathrm{A}}_1^{\mathrm{s}}$ and ${\mathrm{Cp}}_1^{\mathrm{f}}$ were presumed to cause LV_1,_ and those of ${\mathrm{B}}_{10}^{\mathrm{s}},\kern0.5em {\mathrm{Me}}_{10}^{\mathrm{s}},\kern0.5em {\mathrm{A}}_1^{\mathrm{s}},\kern0.5em {\mathrm{Cp}}_1^{\mathrm{f}}$ and VD_10_ to cause LV_overall_, in the formative sense [12]; meanwhile, the extent of final mandibular setback $\left({\mathrm{B}}_{20}^{\mathrm{s}}\right)$ was presumed to be caused by LV_setback_ in the reflective sense (Fig. 2). For the selection of the inner model, where the interactive relationships between LVs should be specified, various possible interactive mapping patterns were tested, and the finalized pathway was chosen based on the *R*-squares of ${R}_{{\mathrm{LV}}_{10\kern0.5em \to \kern0.5em 1}}^2 = 0.9818$ (LV_10_ effect on LV_1_) and ${R}_{{\mathrm{LV}}_{1,\kern0.5em 10,\kern0.5em \mathrm{all}\kern0.5em \to \kern0.5em \mathrm{setback}}}^2 = 0.8731$ (LV_1_, LV_10_, and LV_all_ effects on LV_setback_), with the acceptable GOF value of 0.7236 (Tables 4 and 5). In the finalized PLS-PM, an increase in VD_1_ was found to decrease the absolute value of the final setback amount of the mandible, in the LV_all_ pathway, reflecting the postsurgical physiological responses to both surgery and orthodontic treatment, which in turn can be interpreted as an increase in postoperative mandibular changes.

**Table 3:** Summary statistics for outer model of derived partial-least-square path modeling (PLS-PM)

Latent variables		Weights	Std (weights)	Communality	Redundancy
LV_10_	${\mathrm{B}}_{10}^{\mathrm{s}}$	0.6438	0.991	0.9819	0
	${\mathrm{Me}}_{10}^{\mathrm{s}}$	0.3723	0.973	0.9458	0
LV_overall_	VD_10_	−0.0165	−0.522	0.2729	0.2679
	${\mathrm{B}}_{10}^{\mathrm{s}}$	0.5436	0.982	0.9639	0.9463
	${\mathrm{Me}}_{10}^{\mathrm{s}}$	0.4298	0.964	0.9287	0.9117
	${\mathrm{A}}_1^{\mathrm{s}}$	0.1177	0.176	0.0308	0.055
	${\mathrm{Cp}}_1^{\mathrm{f}}$	0.774	−0.703	0.4944	0
LV_1_	${\mathrm{A}}_1^{\mathrm{s}}$	−0.7146	0.638	0.4068	0
	${\mathrm{Cp}}_1^{\mathrm{f}}$	0.774	−0.703	0.4944	0

**Table 4:** Correlations between latent variables (LVs)

	LV_10_	LV_overall_	LV_1_	LV_setback_
LV_10_	1.000	0.991	0.182	0.898
LV_overall_	0.991	1.000	0.309	0.925
LV_1_	0.182	0.309	1.000	0.409
LV_setback_ (absolute value of final setback of B-point = final setback amount)	0.898	0.925	0.409	1.000

**Table 5:** Summary statistics for inner model of derived PLS-PM

Model summary	
Goodness-of-fit (GoF)	0.7236
LV_overall_	
*R* ^2^	0.9818
Intercept	0
Path loading from LV_10_	0.9908
LV_setback_ (absolute value of final setback of B point = final setback amount)	
*R* ^2^	0.8731
Intercept	0
Path loading from LV_10_	−0.9914
Path loading from LV_overall_	1.9065
Path loading from LV_1_	0.0013

## Discussion

The surgery-first-orthognathic approach is characterized by minimal presurgical orthodontic treatment and orthognathic surgery followed by postsurgical orthodontic treatment. This application has recently been emphasizing its advantages, which include increased patient cooperation, effective compensation, and a shortened treatment period. However, because presurgical orthodontic treatments such as dental decompensation and arch coordination are rarely performed in the surgery-first-orthognathic approach, postsurgical occlusal instability clinically leads to more severe forward mandibular postoperative movement than in conventional surgical orthodontic treatment for skeletal class III deformity.

Our study was triggered by these questions: What variables (CBCT-generated cephalometric measurements) account for the final mandibular setback movements? How does postsurgical occlusal vertical dimension (VD) affect it?

To answer these questions, we applied PLS-PM analysis to the assessment of the treatment outcome for class III SFA cases. From this modeling, we confirmed that the PLS-PM as derived in our study signified the effect of occlusal VD on the extent of final mandibular setback and that an increase in VD leads to a decrease in the absolute amount of the final setback and, ultimately, more severe postsurgical skeletal changes (postsurgical relapse) (Fig. 2).

Tu and co-workers introduced a novel approach to high correlations among the variables using PLS and path-modeling analysis in periodontal research. PLS analysis was used for providing a simple relationship among multiple measures with two or more variables [14, 15]. It could be used for successfully summarizing the inter-correlations among many variables obtained from the analysis, such as the cephalometric analysis data. Lowe and co-workers used PLS analysis to assess the interrelations between obstructive sleep apnea (OSA) outcome variables and computer tomographic, cephalometric, and demographic predictor variables [8].

In the present study, ${\mathrm{B}}_{10}^{\mathrm{s}},\kern0.5em {\mathrm{Me}}_{10}^{\mathrm{s}},\kern0.5em {\mathrm{A}}_1^{\mathrm{s}}$ and ${\mathrm{Cp}}_1^{\mathrm{f}}$, along with VD_10,_ consisted of MVs, which are observable values by definition in the PLS-PM framework (Table 3, Fig. 2), as well as the LVs LV_1_, LV_10_, LV_overall_ and LV_setback_ and their presumed interrelationships (Tables 3 and 4, Fig. 2). Lee et al. built the multiple regression models for the estimation of the final mandibular extent in class III SFA cases. From their study, we re-analyzed these data for this research. From the previous study, the predictors $\left({\mathrm{B}}_{10}^{\mathrm{s}},\kern0.5em {\mathrm{Me}}_{10}^{\mathrm{s}},\kern0.5em {\mathrm{A}}_1^{\mathrm{s}},\kern0.5em {\mathrm{Cp}}_1^{\mathrm{f}},\kern0.5em \mathrm{V}{\mathrm{D}}_{10}\right)$ identified in the general multiple regression model for all 40 patients consisted of the measurement components or, equivalently, the manifest variables (MVs) [9]. The other variables were not significant for predictors in the general model, as Lee and co-workers [9] had already reported. The LVs LV_1_, LV_10_, LV_overall_, and LV_setback_ were not arbitrary but conceptualized the various postsurgical physiologic responses to surgery and orthodontic treatment that ultimately influence the final setback amount of the mandible [11]. These variables were used to evaluate the associations between these variables and the final mandibular setback extent, to determine which variables account for the final mandibular setback extent.

The PLS-PM as derived in our study signified the effect of occlusal vertical dimension on the extent of final mandibular setback (stability of the mandible). As depicted in Fig. 2, an increase in VD_10_ leads to a decrease in the value of LV_overall_, which in turn reduces the value of LV_setback_. The result figure (Fig. 2) explains a decrease in the absolute amount of the final setback and, ultimately, more severe postsurgical skeletal changes (postsurgical relapse). In the model derivation of PLS-PM, the LVs are presumed to represent patterns of physiologic responses after the surgery until the removal of orthodontic appliances, which may or may not be in favor of the expected SFA outcome. Those predictors were manually categorized, based on their temporal dimensions according to either the postsurgical position (*T*_1_) or the amount of surgical movement (∆*T*_1_ − *T*_0_), as LV_1_ or LV_10_, respectively. The underlying physiologic response represented as LV_1_ is likely to involve factors dependent on the postsurgical skeletal positions of the maxilla and proximal segment, apart from the movement of the distal segment, while the physiological response represented as LV_10_ is solely dependent on the amount of horizontal setback of the distal segment of the mandible. Among those presumably related postsurgical factors such as the adaptation or healing process of the masticatory muscles or the change of occlusion due to surgery and orthodontic treatment, the question as to what constitutes LV_1_ and LV_10_ remains and should be studied further in subsequent research.

As for the outer model, the MVs of ${\mathrm{B}}_{10}^{\mathrm{s}}$ and ${\mathrm{Me}}_{10}^{\mathrm{s}}$ were presumed to cause LV_10_, while those of ${\mathrm{A}}_1^{\mathrm{s}}$ and ${\mathrm{Cp}}_1^{\mathrm{f}}$ were presumed to cause LV_1_, and those of ${\mathrm{B}}_{10}^{\mathrm{s}},\ {\mathrm{Me}}_{10}^{\mathrm{s}},\ {\mathrm{A}}_1^{\mathrm{s}},\kern0.5em {\mathrm{Cp}}_1^{\mathrm{f}}$, and VD_10_ to cause LV_overall_, in the formative sense; meanwhile, the extent of final mandibular setback $\left({\mathrm{B}}_{20}^{\mathrm{s}}\right)$ was presumed to be caused by LV_setback_ in the reflective sense (Fig. 2) [11]. For the selection of the inner model, where the interactive relationships between LVs should be specified, various possible interactive mapping patterns were tested, and the finalized pathway was chosen based on the *R*-squares of ${R}_{{\mathrm{LV}}_{10\kern0.5em \to \kern0.5em 1}}^2=\kern0.5em 0.9818$ (LV_10_ effect on LV_1_) and ${R}_{{\mathrm{LV}}_{1,\kern0.5em 10,\kern0.5em \mathrm{all}\kern0.5em \to \kern0.5em \mathrm{setback}}}^2 = 0.8731$ (LV_1_, LV_10_, and LV_all_ effects on LV_setback_), with the acceptable GoF value of 0.7236 (Tables 4 and 5). In the finalized PLS-PM, an increase in VD_10_ was found to decrease the absolute value of the final setback amount of the mandible, in the LV_all_ pathway, as reflective of the postsurgical physiological responses to both surgery and orthodontic treatment, which in turn can be interpreted as an increase in postoperative mandibular changes.

The application of the surgery-first approach (SFA) has recently been reported, with its advantages (increased patient cooperation, effective compensation, and a shortened treatment period) [13–17]. However, orthodontists and oral surgeons often encounter postsurgical occlusal instability, induced from the premature contacts in SFA cases [16–19]. From the current literature and clinical experience, we hypothesized that these postsurgical changes in occlusal vertical dimension might be related to postsurgical skeletal changes (postsurgical relapse). To support the clinical observations and to detect collinearity between measurements, we adopted the partial-least-square path-modeling approach for this study. In clinical dental research, the data might be related to each other. However, we have reviewed the statistical problems such as collinearity. The deductive inference from the clinical research without considering this could sometimes be different from clinical observations, or an unclear conclusion, could be drawn. We attempted to apply the PLS-PM method to support the clinical observations with their interactions. This inference could provide clinicians with clearer cause-and-effect information. Unfortunately, this result was from one of many models with our defined latent variables. As for the shortcomings of our study, first, it was based on a 2-dimensional analysis. Therefore, for improved path modeling, a 3-dimensional analysis of postsurgical skeletal stability should follow this study. At this point, further research is required for better explanations and manifesting variables.

## Conclusions

For the class III SFA, the present study focused on which variables were related and how the occlusal vertical dimension was related to the final mandibular setback movement. To resolve the issue of collinear cephalometric data, we adopted PLS-PM to assess orthodontic treatment. From PLS-PM, we concluded that the more increased the occlusal vertical dimension after surgery, the more changeable the B point position at the posttreatment stage. PLS-PM could be a useful statistical tool to explain the correlation among the variables from cephalometric analysis. However, as sample size increases, PLS becomes less biased. PLS-PM could be used to draw inferences about variables when sample sizes are large. Therefore, a multi-center approach is required to further validate our results for the clinical confirmation of SFA cases.
